# Intrinsic function of the peptidylarginine deiminase PADI4 is dispensable for normal haematopoiesis

**DOI:** 10.1242/bio.059143

**Published:** 2022-06-13

**Authors:** Christine Young, John R. Russell, Louie N. Van De Lagemaat, Hannah Lawson, Christopher Mapperley, Kamil R. Kranc, Maria A. Christophorou

**Affiliations:** 1 MRC Human Genetics Unit, The Institute of Genetics and Molecular Medicine, University of Edinburgh, Edinburgh EH4 2XU, United Kingdom; 2 Centre for Regenerative Medicine, University of Edinburgh, Edinburgh EH16 4UU, United Kingdom; 3 Laboratory of Haematopoietic Stem Cell & Leukaemia Biology, Centre for Haemato-Oncology, Barts Cancer Institute, Queen Mary University of London, London EC1M6BQ, United Kingdom; 4 Epiegetics, Babraham Institute, Cambridge CB22 3AT, United Kingdom

**Keywords:** Peptidylarginine deiminase IV (PADI4), Haematopoietic development, Bone marrow, Differentiation, Regeneration, Ageing

## Abstract

Peptidylarginine deiminases (PADIs) are strongly associated with the development of autoimmunity, neurodegeneration and cancer but their physiological roles are ill-defined. The nuclear deiminase PADI4 regulates pluripotency in the mammalian pre-implantation embryo but its function in tissue development is unknown. PADI4 is primarily expressed in the bone marrow, as part of a self-renewal-associated gene signature. It has been shown to regulate the proliferation of multipotent haematopoietic progenitors and proposed to impact on the differentiation of haematopoietic stem cells (HSCs), suggesting that it controls haematopoietic development or regeneration. Using conditional *in vivo* models of steady state and acute *Padi4* ablation, we examined the role of PADI4 in the development and function of the haematopoietic system. We found that PADI4 loss does not significantly affect HSC self-renewal or differentiation potential upon injury or serial transplantation, nor does it lead to HSC exhaustion or premature ageing. Thus PADI4 is dispensable for cell-autonomous HSC maintenance, differentiation and haematopoietic regeneration. This work represents the first study of PADI4 in tissue development and indicates that pharmacological PADI4 inhibition may be tolerated without adverse effects.

## Abstract (precis)

**Summary:** Inhibition of the pluripotency regulator peptidylarginine deiminase IV (PADI4) does not affect normal haematopoietic development or function, suggesting that targeting it as a therapy can be well tolerated.

## INTRODUCTION

Haematopoietic stem cells (HSCs) possess self-renewal capacity and multi-lineage differentiation potential and are therefore able to replenish all blood cell lineages, sustaining normal and post-injury haematopoiesis. In addition to transcription factors, which directly facilitate or inhibit gene transcription, a central mechanism involved in stem cell fate decisions is the modulation of expression of stem cell and differentiation genes achieved via epigenetic mechanisms such as histone modifications. Indeed, several studies have shown that histone-modifying enzymes are essential for normal haematopoiesis (Chan et al., 2003; Loizou et al., 2009; Ou et al., 2011; Thieme et al., 2013; Wang et al., 2014; Liu et al., 2015; Greenblatt et al., 2016).

Peptidylarginine deiminase (PADI, or PAD) enzymes catalyse citrullination, the post-translational conversion of protein arginine residues to the non-coded amino acid citrulline. The five PADI family members are structurally similar and likely to operate via common regulatory mechanisms (Arita et al., 2004; Slade et al., 2015), but they show varying tissue distributions and sub-cellular localisations, suggesting that they have specific organismal roles. PADI deregulation is strongly associated with disease development (Wang and Wang, 2013; Lewis and Nacht, 2016). Aberrantly high citrullination levels underlie the development of autoimmunity and are strongly associated with the development neurodegeneration and cancer (Suzuki et al., 2003; Musse et al., 2008; Zhang et al., 2011; Wang and Wang, 2013; Witalison et al., 2015; Yuzhalin et al., 2018). For this reason significant efforts have been devoted towards generating inhibitors against PADIs (Lewis and Nacht, 2016), necessitating a better understanding of their physiological functions.

The nuclear deiminase PADI4 citrullinates core and linker histones and has well-established roles in the regulation of gene transcription and chromatin compaction (Cuthbert et al., 2004; Wang et al., 2004; Tanikawa et al., 2009; 2018; Guo and Fast, 2011; Zhang et al., 2011; Stadler et al., 2013; Wang and Wang, 2013; Christophorou et al., 2014). We previously showed that PADI4 regulates the establishment of pluripotency during mammalian pre-implantation embryo development and cell reprogramming (Christophorou et al., 2014), however it is not known whether PADI4 plays a role in tissue development. Out of all mammalian tissues, *Padi4* is most highly expressed in the bone marrow (BM) and peripheral blood (PB) and is one of the top 50 genes associated with self-renewal, as determined by the fact that it is expressed in HSCs, downregulated upon differentiation to multi-lineage progenitors, but upregulated in leukaemia stem cells (Krivtsov et al., 2006). More recent studies showed that haematopoietic multipotent progenitor cells from constitutive *Padi4*-null mice exhibit increased proliferation (Nakashima et al., 2013). Additional work showed that PADI4 regulates the expression of c-Myc and acts as a co-activator of translocated in leukaemia 1 (Tal1) (Nakashima et al., 2013; Kolodziej et al., 2014). c-Myc and Tal1 are critical transcriptional regulators in the haematopoietic system, suggesting that PADI4 regulates the differentiation of haematopoietic stem or progenitor cells (Kolodziej et al., 2014). Taken together, these findings suggest that PADI4 functions in the regulation of haematopoiesis.

To understand the role of PADI4 in normal haematopoiesis, HSC maintenance, haematopoietic regeneration and ageing, we carried out a systematic analysis using mouse models of constitutive and inducible *Padi4* deletion from the haematopoietic system. We demonstrate that HSCs do not require intrinsic PADI4 to self-renew, sustain long-term multilineage haematopoiesis or respond to haematopoietic injury. Moreover, by investigating long-term consequences of *Padi4* deletion, we show that *Padi4* loss does not lead to HSC exhaustion or premature ageing.

## RESULTS

### *Padi4* ablation does not significantly affect steady-state haematopoiesis

**Fig. 1.**
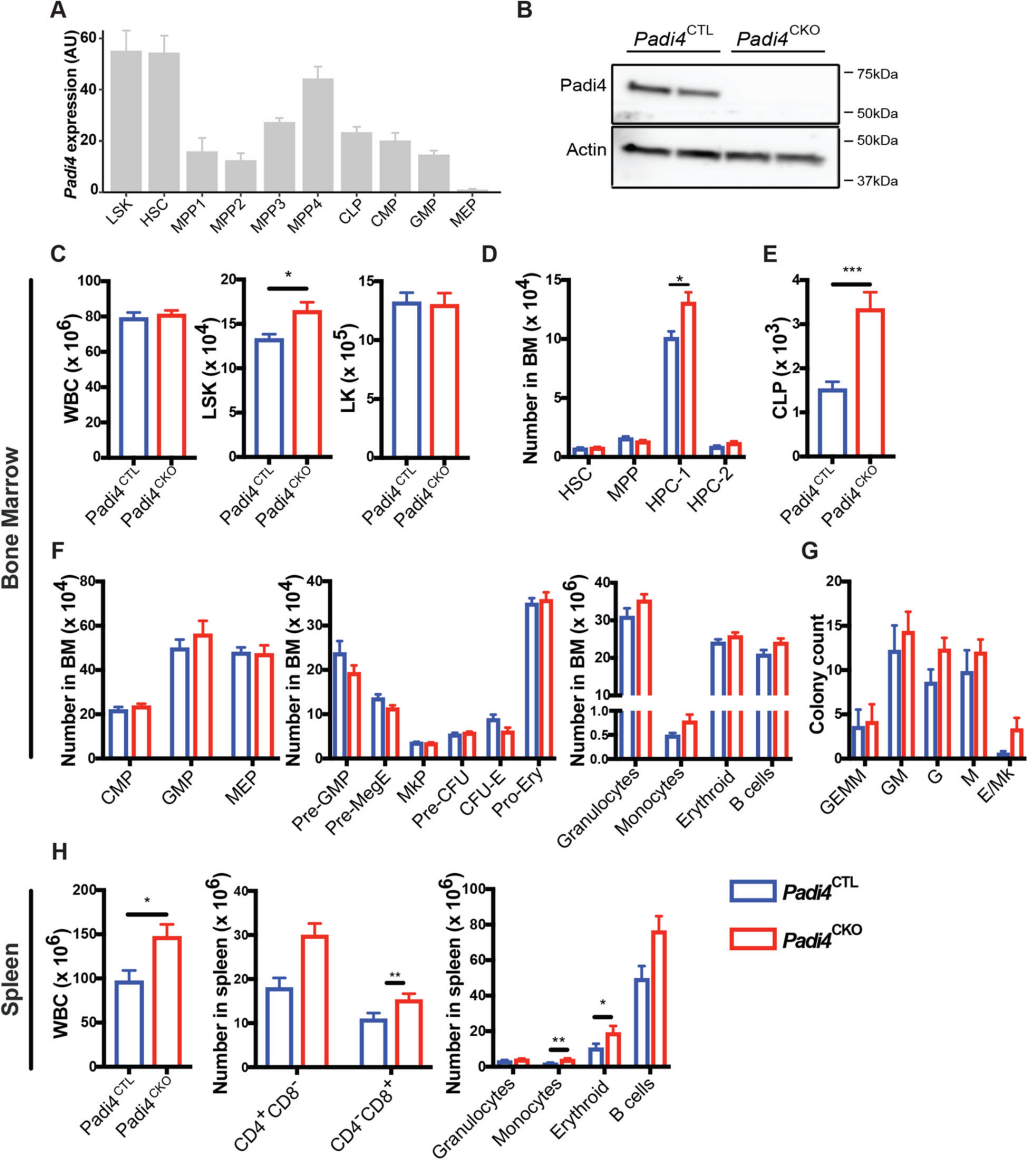
**Haematopoiesis-specific deletion of PADI4 has no major impact on steady-state haematopoiesis.** (A) Padi4 expression derived from five bulk sequencing studies and batch corrected, with each study contributing to an average of four populations. (B) Immunoblot analysis of mouse PADI4 in total BM extracts from *Padi4*^CTL^ and *Padi4*^CKO^ mice. Actin presented as a loading control. (C–H) Immunophenotypic analysis of bone marrow from 8–12-week-old mice; total number of (C) WBC, LSK, and LK cells (D) HSC, MPP, HPC-1 and HPC-2 cells. *Padi4*^CTL^, *n*=9; *Padi4*^CKO^, *n*=9. (E,F) Total number of lymphoid, myeloid and erythroid progenitor cells. (E) CLP, (F) CMP, GMP, MEP, Pre-GMP, Pre-MegE, MkP, Pre-CFU, CFU-E, Pro-Ery and differentiated cell populations (Granulocytes, Monocytes, Erythroid and B cells). *Padi4*^CTL^, *n*=9; *Padi4*^CKO^, *n*=9. (G) CFC assay with BM cells. *Padi4*^CTL^, *n*=5; *Padi4*^CKO^, *n*=6. (H) Immunophenotypic analysis of spleen from 8–12-week-old mice; total number of WBC, T cells and differentiated cell populations (granulocytes, monocytes, erythroid and B cells). *Padi4*^CTL^, *n*=9; *Padi4*^CKO^, *n*=9. All data are mean±s.e.m. *, *P*<0.05; **, *P*<0.01; ***, *P*<0.001, ****, *P*<0.0001 (Mann–Whitney *U*-test).

Mammalian PADI enzymes exhibit tissue specific expression and PADI4 is expressed mainly the BM and in peripheral blood neutrophils, eosinophils and monocytes (Nakashima et al., 2002; Vossenaar, 2004; Krivtsov et al., 2006; Nakashima et al., 2013). Global transcriptomic analyses of sorted BM cell populations shows that *Padi4* is expressed most highly in haematopoietic stem and progenitor cells (Fig. 1A), while PADI4 protein is readily detectable in the BM (Fig. 1B). To determine the functional significance of PADI4 in steady-state haematopoiesis and HSC self-renewal, we deleted *Padi4* specifically from the haematopoietic system using the *Vav-iCre* system. *Vav-iCre* mice (de Boer et al., 2003) constitutively express the codon-improved Cre (iCre) (Shimshek et al., 2002) driven by the *Vav* regulatory elements (Ogilvy et al., 1999), resulting in haematopoietic-specific gene deletion shortly after the emergence of definitive HSCs (Chen et al., 2009) and ensuring recombination in all HSCs (Buza-Vidas et al., 2011; Paris et al., 2019). We bred these mice to *Padi4^fl/fl^* mice (Hemmers et al., 2011), in which *Padi4* exons 9 and 10 are flanked by *loxP* sites. These exons contain aspartate 352, which is part of the active site, as well as four additional residues (Q351, E353, E355, D371), which are essential for Ca^2+^ binding and activation of the enzyme (Arita et al., 2004). The resulting *Padi4^fl/fl^;Vav-iCre* mice (referred to as *Padi4*^CKO^, for *Padi4* conditional knockout, hereafter) completely lack PADI4 protein expression in the BM (Fig. 1B). These mice were compared to *Padi4*^fl/fl^ mice (referred to as *Padi4*^CTL^, for *Padi4* control, hereafter) in all subsequent analyses. *Padi4*^CKO^ and *Padi4*^CTL^ mice showed normal Mendelian distribution, had comparable survival and did not display any obvious defects. To enumerate cells at different levels of the haematopoietic differentiation hierarchy, we next carried out immunophenotypic analyses of *Padi4*^CKO^ mice. In agreement with a previous report (Nakashima et al., 2013), *Padi4*^CKO^ mice had increased numbers of Lin^−^Sca-1^+^c-Kit^+^ (LSK) cells but similar numbers of total white blood cells (WBC) and lineage restricted myeloid and erythroid Lin^−^Sca-1^−^c-Kit^+^ (LK) progenitor cells compared to *Padi4*^CTL^ mice (Fig. 1C). Further analysis of the LSK compartment showed normal numbers of LSKCD48^−^CD150^+^ HSCs, LSKCD48^−^CD150^−^ multipotent progenitors (MPPs), LKSCD48^+^CD150^+^ primitive haematopoietic progenitors (HPC-2) and an increase in LSKCD48^+^CD150^−^ haematopoietic progenitor cell-1 (HPC-1) population in *Padi4*^CKO^ mice compared to *Padi4*^CTL^ mice (Fig. 1D). We observed an increase in common lymphoid progenitors (Lin^−^c-Kit^lo^Sca^lo^IL7Rα^+^cells) in *Padi4*^CKO^ mice compared to *Padi4*^CTL^ mice (Fig. 1E). However, the numbers of myeloid and erythroid progenitors, CD11b^+^Gr1^−^ and CD11b^+^Gr1^+^ differentiated myeloid cells, CD19^+^ B cells and Ter119^+^ erythroid cells in the BM were comparable between *Padi4*^CKO^ and *Padi4*^CTL^ mice (Fig. 1F). These results were mirrored in *in vitro* colony forming cell (CFC) assays where there was no difference in colony counts between the two genotypes (Fig. 1G). In addition, numbers of thymic T-cells were unaffected (Fig. S1A). PB analysis showed that the numbers of circulating blood cells and haemoglobin parameters were completely unaffected by *Padi4* deletion (Fig. S1B). Analysis of the spleens showed a modest increase in differentiated cells in *Padi4*^CKO^ mice with an overall increase in WBC counts, indicating extramedullary haematopoiesis (Fig. 1H). In conclusion, despite mild extramedullary haematopoiesis, *Padi4* deletion has no major impact on BM steady-state haematopoiesis.

### *Padi4* is dispensable for HSC maintenance

**Fig. 2.**
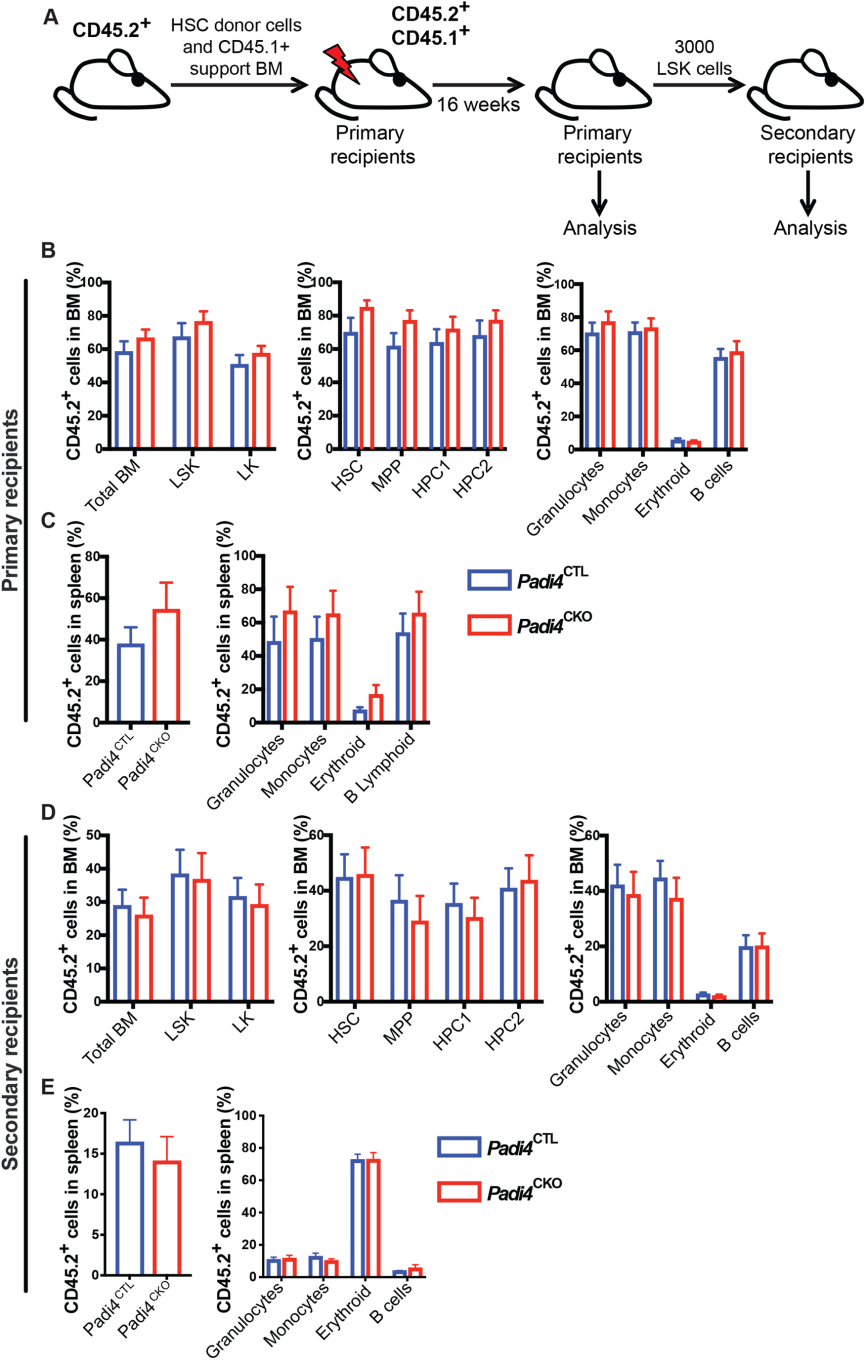
**Haematopoiesis-specific deletion of PADI4 has no impact on BM reconstitution potential following serial transplantations.** (A) Experimental design for BM transplantation experiments. 200 CD45.2^+^ BM HSCs from C57BL/6 *Padi4*^CTL^ or *Padi4*^CKO^ mice were transplanted into primary recipient mice and monitored for 16 weeks. Following this, a cohort of mice were sacrificed for analysis at 16 weeks post-transplantation and bone marrow was transplanted to secondary recipients. (B) Percentage of donor-derived CD45.2_+_ cells in total BM, LSK, LK, HSC, MPP, HPC-1, HPC-2 and differentiated cell populations (granulocytes, monocytes, erythroid and B cells). *Padi4*^CTL^, *n*=14; *Padi4*^CKO^, *n*=13. (C) Contribution of donor-derived CD45.2^+^ cell population to total spleen WBC count and differentiated cell populations of primary recipients. *Padi4*^CTL^, *n*=6; *Padi4*^CKO^, *n*=6. (D,E) Secondary recipient mice were transplanted with 3000 sorted CD45.2^+^ BM LSK cells from primary recipients euthanised at 16 weeks. (D) Percentage of donor-derived CD45.2^+^ cells in total BM, LSK, LK, HSC, MPP, HPC-1, HPC-1 and differentiated cell lineages (granulocytes, monocytes, erythroid and B cells). *Padi4*^CTL^, *n*=19; *Padi4*^CKO^, *n*=19. Two to four donors were used per genotype. (E) Contribution of donor-derived CD45.2^+^ cell population to spleen WBC and differentiated cells of secondary recipients. *Padi4*^CTL^, *n*=19; *Padi4*^CKO^, *n*=19. All data are mean±s.e.m. *P<0.05; **P<0.01; ***P<0.001, ****P<0.0001 (Mann–Whitney *U*-test).

To assess the requirement for *Padi4* in HSC maintenance, we performed competitive HSC transplantation assays. CD45.2^+^LSKCD48^−^CD150^+^ HSCs sorted from *Padi4*^CKO^ and control (*Padi4*^CTL^) mice were competitively transplanted into lethally irradiated wild-type syngeneic CD45.1^+^/CD45.2^+^ recipients (Fig. 2A). Peripheral blood analysis showed no difference in CD45.2^+^ donor-derived chimerism in primary recipients of the *Padi4*^CKO^ HSCs when compared to recipients of *Padi4*^CTL^ HSCs (Fig. S2A). BM analysis at 16 weeks post-transplantation showed that HSCs of both genotypes efficiently reconstituted long-term multi-lineage haematopoiesis, while donor-derived cells contributed equally to BM HSC and primitive cell compartments of the primary recipient mice (Fig. 2B). No difference in CD45.2^+^ cell engraftment was found in the spleen of recipient mice and both *Padi4*^CTL^ and *Padi4*^CKO^ donor derived cells contributed equally to differentiated cell populations in the spleen (Fig. 2C). Moreover, *Padi4*^CKO^ LSK cells sustained long-term BM reconstitution in secondary recipients comparably to *Padi4*^CTL^ LSK cells (Fig. 2D), while equal engraftment of CD45.2^+^ cell engraftment was observed in spleen (Fig. 2E) of the secondary recipients. In addition, no difference in CD45.2^+^ cell engraftment was found in PB (Fig. S2B). These experiments revealed that HSCs do not require *Padi4* to self-renew and sustain long-term multi-lineage haematopoiesis upon transplantation.

### Acute *Padi4* deletion does not affect haematopoietic development

**Fig. 3.**
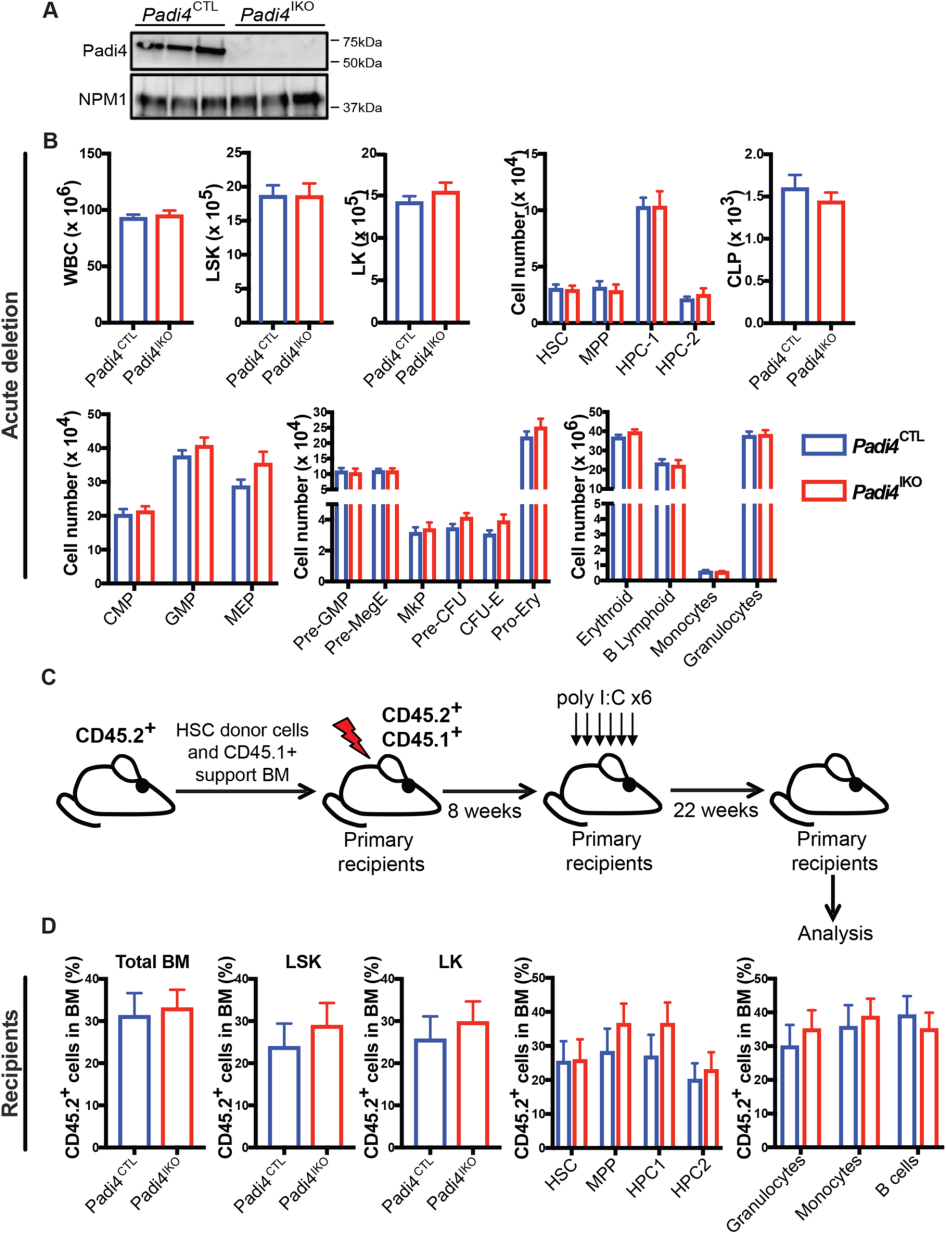
**Acute deletion of *Padi4* in adult HSCs.** *Padi4*^CTL^ and *Padi4*^IKO^ mice received 6x intraperitoneal (IP) injections of Poly I:C to induce deletion of Padi4. (A) Immunoblot analysis of mouse PADI4 in total BM extracts from *Padi4*^CTL^ and *Padi4*^IKO^ mice. Nucleophosmin (NPM1) presented as a loading control. (B) Immunophenotypic analysis performed 4 weeks following the final injection. Total number of: WBC, LSK, LK, HSC, MPP, HPC-1 and HPC-2 cells; myeloid, erythroid and lymphoid progenitor cells: GMP, MEP, CMP, CLP Pre-GMP, Pre-MegE, MkP, Pre-CFU, CFU-E, Pro-Ery; differentiated granulocytes, monocytes, B cells and erythroid cells in the BM. *n*=5–8 per genotype. (C,D) Transplantation of BM after acute *Padi4* deletion. (C) Schematic of experimental procedure for transplantation of *Padi4*^CTL^ and *Padi4*^IKO^ BM cells. 2×10^5^ unfractionated CD45.2^+^ BM cells from untreated *Padi4*^CTL^ and *Padi4*^IKO^ C57BL/6 (8–12-week-old) mice were mixed with 2×10^5^ CD45.1^+^ WT BM cells and transplanted into lethally irradiated CD45.1^+^/CD45.2^+^ recipients. 8 weeks after transplantation, the recipients received six doses of Poly I:C and the BM was analysed 22 weeks after the last injection. (D) Percentage of donor-derived CD45.2^+^ cells in the BM of recipient mice: LSK, LK, HSC, MPP, HPC-1, HPC-1 and differentiated cell lineages (granulocytes, monocytes and B cells). *n*=15–21 recipients per genotype. *n*=3–4 donors per genotype. All data are mean±s.e.m. *P<0.05; **P<0.01; ***P<0.001, ****P<0.0001 (Mann–Whitney *U*-test).

Given that *Vav-iCre* recombines in the embryo soon after the emergence of definitive HSCs, it is possible that *Vav-iCre*-mediated *Padi4* deletion may activate compensatory mechanisms that bypass *Padi4* deficiency. To rule this out, we examined HSC maintenance following acute *Padi4* ablation. We generated *Padi4^fl/fl^;Mx1-Cre* mice (referred to as *Padi4*^IKO^, for *Padi4* inducible knockout, hereafter) in which efficient recombination in the BM is induced by treatment with Poly I:C (Kühn et al., 1995) and leads to complete loss of PADI4 protein (Fig. 3A). *Padi4*^IKO^ and *Padi4*^CTL^ mice received six injections of 300 μg Poly I:C, on every other day, and were euthanised and analysed 4 weeks following the final administration. We found that acute deletion of *Padi4* had no effect on any BM cell compartment analysed (Fig. 3B). To test whether acute *Padi4* deletion affects post-transplantation haematopoietic reconstitution, we transplanted CD45.2^+^ unfractionated BM cells from *Padi4*^IKO^ or *Padi4*^CTL^ mice with support CD45.1^+^ BM cells to lethally irradiated recipient mice. Following efficient CD45.2^+^ cell engraftment (8 weeks post-transplantation), the mice received six doses of Poly I:C resulting in efficient *Padi4* deletion in donor-derived CD45.2^+^ cells (Fig. 3C). The contribution of CD45.2^+^ cells to the PB of the recipients was quantified at 4, 8, 12, 18 and 22 weeks post-transplantation and no differences were observed (Fig. S3). The donor-derived contribution of CD45.2^+^ cells to BM cell compartments of the recipients, including total WBC, LSK, LK and HSCs, was similar regardless of the genotype of transplanted CD45.2^+^ cells, as was the CD45.2^+^ cell contribution to differentiated cell lineages in the BM (Fig. 3D).

### *Padi4* is dispensable for the regenerative capacity of HSCs

**Fig. 4.**
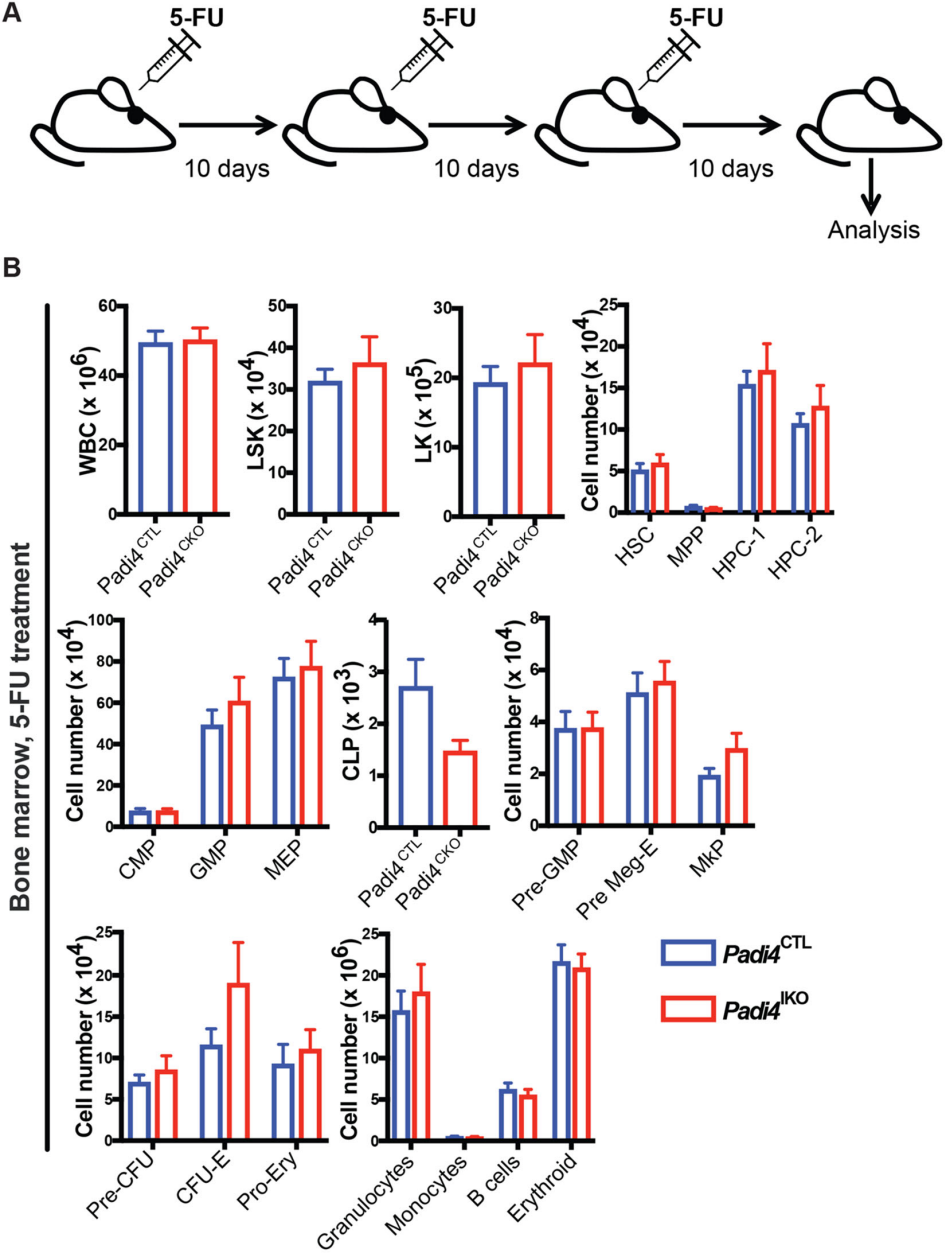
**Assessment of *Padi4* deletion the response to haematopoietic injury.** (A) Experimental design of haematopoietic injury approach. *Padi4*^CTL^ and *Padi4*^CKO^ mice received a 3x 5-FU injections at 150 mg/kg 10 days apart and were analysed 10 days after the last administration. (B) Immunophenotypic analysis of *Padi4*^CTL^ and *Padi4*^CKO^ mice was performed 10 days after the final dose of 5-FU. Total number of cells in BM: WBC, LSK, and LK; HSC, MPP, HPC-1 and HPC-2 cells; myeloid, erythroid and lymphoid progenitor cells: CMP, GMP, MEP, CLP, Pre-GMP, Pre-MegE, MkP, Pre-CFU, CFU-E, Pro-Ery; differentiated granulocytes, monocytes, B cells, erythroid cells. *Padi4*^CTL^, *n*=21; *Padi4*^CKO^, *n*=18. All data are mean±s.e.m. *P<0.05; **P<0.01; ***P<0.001, ****P<0.0001 (Mann–Whitney *U*-test).

To test the role of *Padi4* in HSC regenerative capacity upon haematopoietic injury, we treated adult (8–12 week) *Padi4*^CKO^ and *Padi4*^CTL^ mice with 5-fluorouracil (5-FU). Mice received three injections of 5-FU 10 days apart and BM was analysed 10 days following the final dose (Fig. 4A). We observed no difference in numbers of HSCs, LSK, LK cell compartments or differentiated cells in the BM of *Padi4*^CKO^ mice compared to *Padi4*^CTL^ mice (Fig. 4B; Fig. S4). Therefore, PADI4 is dispensable for the ability of HSCs to respond to haematopoietic stress.

### *Padi4* ablation does not lead to HSC exhaustion upon ageing

**Fig. 5.**
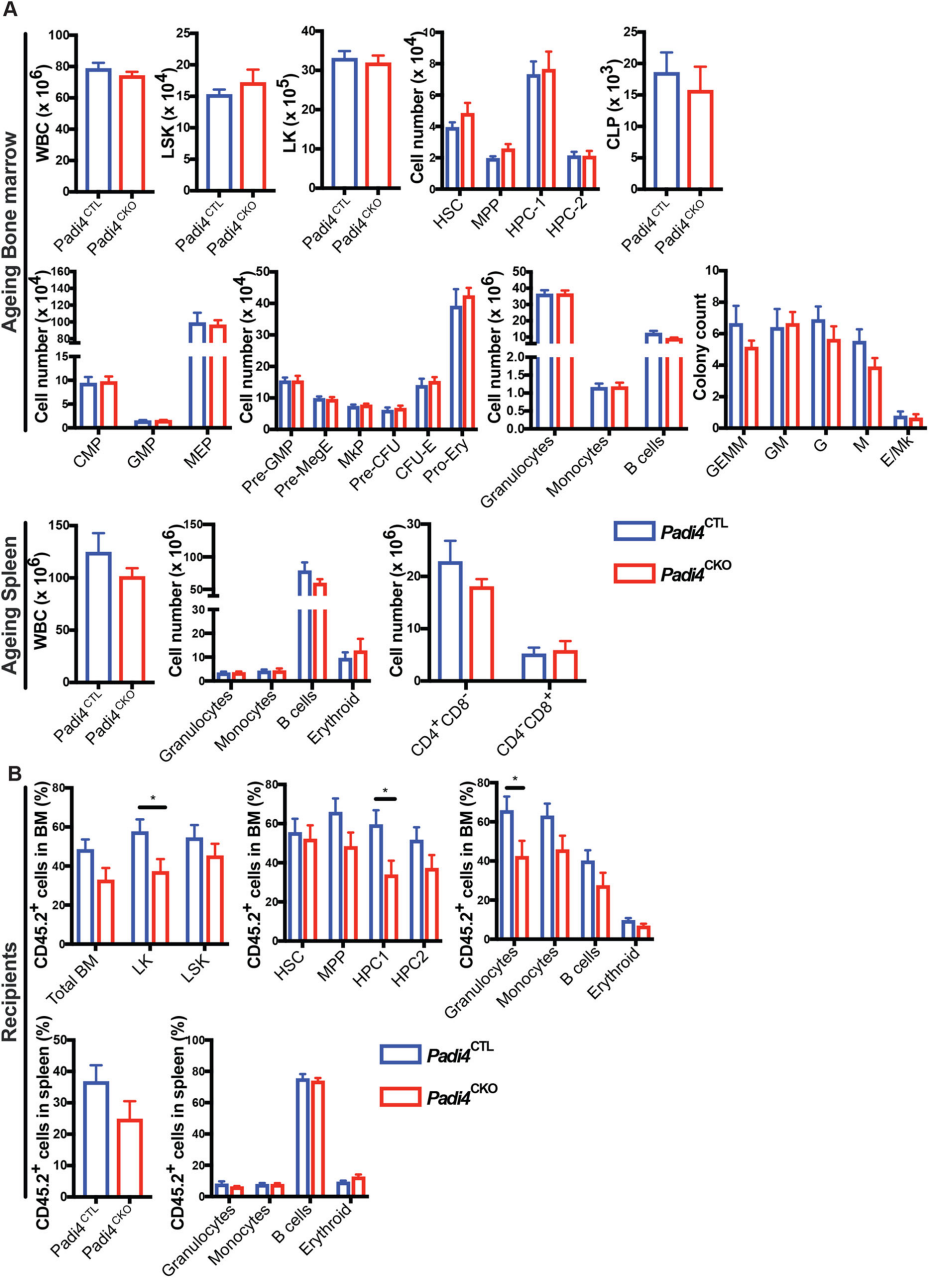
**Assessment of *Padi4* deletion on ageing of HSCs.** (A) Immunophenotypic analysis in BM of *Padi4*^CTL^ and *Padi4*^CKO^ mice aged for 1 year. WBC, LSK, LK; HSC, MPP, HPC-1 and HPC-2 cells; myeloid, erythroid and lymphoid progenitor cells: CMP, GMP, MEP, CLP, Pre-GMP, Pre-MegE, MkP, Pre-CFU, CFU-E, Pro-Ery; differentiated granulocytes, monocytes, B cells, erythroid cells. CFC assay with aged BM samples. Total number of cells in spleen; WBC, granulocytes, monocytes, B cells, erythroid cells and T cells. *Padi4*^CTL^, *n*=9; *Padi4*^CKO^, *n*=8. (B) 200 CD45.2^+^ BM HSCs from 1-year-old mice were transplanted to primary recipient mice and monitored for 24 weeks following which immunophenotypic analysis was performed on BM and spleen. Contribution of donor derived CD45.2^+^ cells to the granulocyte, monocyte, B cell and T cell population in PB. *Padi4*^CTL^, *n*=20; *Padi4*^CKO^, *n*=16. Percentage of donor-derived CD45.2^+^ cells in total BM, LSK, LK, HSC, MPP, HPC-1, HPC-1 and differentiated cell lineages (granulocytes, monocytes, B cells and erythroid cells). Contribution of donor-derived CD45.2^+^ cell population to total spleen WBC count and differentiated cell populations. *Padi4*^CTL^, *n*=17; *Padi4*^CKO^, *n*=15. Three to four donors were used per genotype. All data are mean±s.e.m. *P<0.05; **P<0.01; ***P<0.001, ****P<0.0001 (Mann–Whitney *U*-test).

To investigate the long-term effects of *Padi4* deletion in the haematopoietic system, we carried out immunophenotypic analyses of *Padi4*^CKO^ mice aged up to 1 year, as well as BM reconstitution experiments using HSCs from these aged mice (Fig. 5A,B). Analysis of BM and spleens of 1-year-old *Padi4*^CKO^ mice and *Padi4*^CTL^ mice showed no difference in cell counts of total WBC, primitive LSK compartments, LK progenitor populations or differentiated cell populations (Fig. 5A). In addition, *in vitro* CFC assays using aged BM from *Padi4*^CKO^ and *Padi4*^CTL^ mice also showed no difference in CFC colony count between the two genotypes (Fig. 5A). In fact, the differences observed in young mice (Fig. 1C,D,E,H) were not observed upon ageing. To assess the self-renewal potential of aged *Padi4*^CKO^ HSCs, we transplanted sorted HSCs from 1-year-old *Padi4*^CKO^ and *Padi4*^CTL^ mice into primary recipients and analysed the bone marrow of the recipients at 36 weeks post-transplantation. Mice transplanted with *Padi4*^CKO^ HSCs showed a small decrease in the contribution of donor derived CD45.2^+^ cells to the LK and HPC1 progenitor populations as well as a decrease in differentiated granulocytes (Fig. 5B). No differences in the ability of *Padi4*^CKO^ HSCs to contribute to spleen or peripheral blood cells was found (Fig. 5B; Fig. S5). Taken together, these results show that *Padi4* loss does not significantly affect long-term cell-autonomous HSC maintenance and normal haematopoiesis.

## DISCUSSION

This study demonstrates that the nuclear peptidylarginine deiminase PADI4, which is most highly expressed in the bone marrow and peripheral blood, is not required for cell-autonomous steady-state haematopoiesis, long-term self-renewal of HSCs, efficient reconstitution of multi-lineage haematopoiesis in serial transplantation assays or response of HSCs to haematopoietic injury. Histone citrullination is antagonistic to histone arginine methylation (Cuthbert et al., 2004; Wang et al., 2004), a modification that has established roles in the regulation of haematopoiesis (Kolodziej et al., 2014; Greenblatt et al., 2016) and PADI4 was previously shown to influence gene expression by counteracting arginine methylation in leukaemia cells (Kolodziej et al., 2014). As a result, PADI4 was proposed to modulate lineage specification of haematopoietic stem and progenitor cells (Kolodziej et al., 2014). However, our *in vivo* data show that genetic *Padi4* deletion does not have a significant effect on haematopoiesis. PADI4 is widely deregulated in cancer development (Chang and Han, 2006). Collectively, these findings suggest that deregulated, but not physiological, PADI4 levels can interfere with histone arginine methylation-regulated gene regulation in the haematopoietic system. A previous study conducted using *Padi4*-null mice (Nakashima et al., 2013) suggested that PADI4 regulates the proliferation of multipotent stem cells in the BM, as such mice showed increased numbers of LSK cells. Our experiments using haematopoiesis-specific deletion of *Padi4* replicate this phenotype but show that it does not translate into measurable effects in any aspect of haematopoiesis.

The results presented here have significant implications for biomedicine. PADI4 is upregulated in leukaemia stem cells during the development of acute myeloid leukaemia (Fig. 1A; Krivtsov et al., 2006). Furthermore, it was shown to act as a co-activator of Tal1, a key transcriptional regulator in the development of T-cell acute lymphoblastic leukaemia (Chen et al., 1990; Kolodziej et al., 2014). PADI4 is therefore a promising target for the treatment of leukaemia and our results indicate that targeting it may offer a therapeutic window. Most pertinently, significant efforts are currently underway to develop PADI4 inhibitors for the treatment of rheumatoid arthritis, systemic lupus erythematosus, atherosclerosis, thrombosis, sepsis, irritable bowel syndrome, and colon cancer (Lewis and Nacht, 2016). The fact that ablation of *Padi4* does not lead to impairment in the functions of the haematopoietic system, where it is most highly expressed, suggests that systemic PADI4 inhibition may be administered without adverse effects.

## MATERIALS AND METHODS

### Mice

All experiments on animals were performed under UK Home Office authorisation. All mice were of C57BL/6 genetic background. *Padi4*^fl/fl^ mice (Hemmers et al., 2011) were a kind gift from the Mowen lab. *Vav-iCre* and *Mx1-Cre*, have been described previously (Kühn et al., 1995; de Boer et al., 2003; Vukovic et al., 2015). All transgenic and knockout mice were CD45.2^+^. Congenic recipient mice were CD45.1^+^/CD45.2^+^. Sex-matched 8- to 12-week-old mice were used throughout. All experiments on mice were performed under University of Edinburgh's Veterinary oversight with UK Home Office authorization.

### Flow cytometry

All BM cells were prepared and analysed as described previously (Kranc et al., 2009; Mortensen et al., 2011; Guitart et al., 2013; 2017; Vukovic et al., 2015). BM cells were isolated by crushing tibias and femurs using a pestle and mortar. Cell suspensions were passed through a 70 μm strainer. PB was collected in EDTA coated microvettes. Spleen and thymus were homogenised and passed through a cell strainer. Single cell suspensions were incubated with Fc block and then stained with antibodies. For HSC cell analyses, unfractionated BM cells were stained with a lineage marker cocktail containing biotin-conjugated anti-CD4, anti-CD5, anti-CD8a, anti-CD11b, anti-B220, anti-Gr-1 and anti-Ter119 antibodies together with APC-conjugated anti-c-Kit, FITC-conjugated anti-Sca-1, PE-conjugated anti-CD48 and PE-Cy7-conjugated anti-CD150 antibodies. Biotin-conjugated antibodies were then stained with Pacific Blue-conjugated streptavidin. For pan-lineage progenitor cell staining, cells were stained with the lineage marker cocktail described above together with APC-conjugated anti-c-kit, PE-Cy7-conjugated anti-Sca-1, BV-421-conjugated anti-CD127, FITC-conjugated anti-CD34, PE-conjugated anti-CD135 and APC-Cy7-conjugated anti-CD16/32. For myeloid/T lymphoid restricted progenitors, cells were stained with a lineage marker cocktail containing biotin-conjugated anti-CD4, anti-CD5, anti-CD8a, anti-Mac-1, anti-B220, anti-CD19 and anti-Gr-1 together with BV-510-conjugated anti-c-kit, Pacific Blue-conjugated anti-Sca-1, PE-Cy7-conjugated anti-CD150, APC-Cy7-conjugated anti-CD16/32, APC-conjugated anti-CD41, PE-conjugated anti-CD105 and FITC-conjugated anti-Ter119. Biotin-conjugated antibodies were then stained with PerCP-conjugated streptavidin. For analyses of differentiated cells, spleen, BM or PB cell suspensions were stained with APC-Cy7-conjugated anti-CD19 antibody for B cells; Pacific Blue-conjugated anti-CD11b and PE-Cy7-conjugated anti-Gr-1 for myeloid cells; APC-conjugated anti-CD8 antibodies and PE-conjugated anti-CD4 antibodies for T cell analysis (spleen and PB); FITC-conjugated anti-Ter119 and PE-conjugated anti-CD71 (BM).

To distinguish CD45.2^+^-donor derived cells in the BM and spleen of transplanted mice, BV711-conjugated anti-CD45.1 and Pacific Blue-conjugated anti-CD45.2 antibodies were used. For HSC staining in transplanted mice, the remainder of the staining was as described above. For analyses of differentiated cells in BM and spleen of transplanted mice, myeloid cells were stained with PE-conjugated anti-CD11b, PE-Cy7-conjugated anti-Gr-1 and FITC-conjugated anti-Ter119 for erythroid cells. B Lymphoid cells were stained with APC-Cy7-conjugated anti-CD19. PB of transplanted mice was stained with FITC-conjugated anti-CD45.1, Pacific Blue-conjugated anti-CD45.2, PE-conjugated anti-CD4 and CD8a, PE-Cy7-conjugated anti-Gr-1, APC-conjugated anti-CD11b, and APC-Cy7-conjugated anti-CD19.

The catalogue numbers, clone numbers and manufacturer information for the above antibodies is provided in Table S1.

Flow cytometry analyses were performed using a LSRFortessa (BD Biosciences). Cell sorting was performed on a FACSAria Fusion (BD Biosciences).

### CFC assays

CFC assays were carried out using MethoCult^TM^ M3434 (STEMCELL Technologies) methylcellulose medium (Lawson et al., 2021a,b). Two technical replicates were used per each biological replicate in each experiment. BM cells were plated for 10 days before colony types were identified and counted.

### Blood profiling

Blood was collected via cardiac puncture into an EDTA coated microvette and analysed on a Celltaq Haematology analyser (Nihon Kohden).

### Syngeneic transplantation assays

CD45.1^+^/CD45.2^+^ C57BL/six recipient mice were lethally irradiated using a split dose of 11 Gy (two doses of 5.5 Gy administered at least 4 h apart) at an average rate of 0.58 Gy/min using a Cesium 137 GammaCell 40 irradiator (Mapperley et al., 2020). For primary transplantations 200 LSKCD48^−^CD150^+^ HSCs (per recipient) sorted from BM of the donor mice were mixed with 200,000 unfractionated support CD45.1^+^ wild-type BM cells and transferred into lethally irradiated CD45.1^+^/CD45.2^+^ recipients. For secondary transplantations 2000-3000 CD45.2^+^ LSK cells sorted from BM of primary recipients were mixed with 200,000 unfractionated support CD45.1^+^ wild-type BM cells and re-transplanted. For all except ageing experiments, primary and secondary recipients were culled and analysed 16–20 weeks post-transplant.

### Poly I:C administration

Both *Padi4*^CTL^ and *Padi4*^IKO^ *(Padi4;Mx1-Cre)* transgenic and CD45.1^+^/CD45.2^+^ C57BL/six recipient mice were injected intraperitoneally with poly I:C. *Padi4^CTL^ and Padi4*^IKO^ mice received six injections of 300 μg Poly I:C, on every other day. Mice were culled and analysed 1 month following the final administration. Recipient mice received one injection every other day with 300 μg Poly I:C (GE Healthcare) for a total of six doses starting 8 weeks after transplantation as previously described (Kranc et al., 2009; Guitart et al., 2013; 2017).

### 5-FU administration

Both *Padi4^CTL^* and *Padi4^CKO^* transgenic mice were injected intraperitoneally with 5-FU. Mice were weighed on the day of administration and received three injections of 150 mg/kg 10 days apart. Mice were culled and analysed 10 days following the final administration.

### Western blotting

Proteins extracted from *Padi4^CTL^* and *Padi4^CKO^* were subjected to SDS–PAGE (4–20% Mini-PROTEAN® TGX™ Precast gel, Bio-Rad) and then transferred onto a nitrocellulose membrane. Membranes were blocked in 5% BSA-TBST (TBS with 0.1% Tween20) and probed with anti-Padi4 (Abcam, ab214810, 1:1000, O/N at 4°C) and anti-Actin (Santa Cruz Biotechnology, sc-1616, 1:500, O/N at 4°C). After incubation with appropriate horseradish peroxidase-coupled secondary antibody, proteins were detected with SuperSignal™ West Pico PLUS Chemiluminescent Substrate (Thermo Fisher Scientific) and acquired on the ImageQuant LAS (GE Healthcare Life Sciences).

### Transcriptomic analyses

Expression data were derived from multiple bulk-sequencing datasets from ArrayExpress and NCBI GEO. Each study, identified by accession number, contributed data to multiple populations as follows: E-MTAB-1963 (LSK, CMP); E-MTAB-3079 (LSK, CLP, CMP, GMP); E-MTAB-2262 (HSC, MPP1, MPP2, MPP3, MPP4); GSE116177 (LSK, CLP, CMP, GMP); GSE125846 (HSC, CMP, GMP, MEP). Data were aligned to the GRCm38 mouse genome using hisat2, and inter-study batch correction was performed using the batchelor package in R, after which FPKM expression values were computed and plotted.

### Genotyping

DNA was extracted from bone marrow taken from recipient mice transplanted with *Padi4*^CTL^, *Padi4*^CKO^ or *Padi4*^IKO^ mice. PCR was performed using primers specific for *Padi4* deletion. Forward primer: 5′-CAG GAG GTG TAC GTG TGC A-3′. Reverse primer: 5′-AGT CCA GCT GAC CCT GAA C-3′. Expected band sizes: wild-type *Padi4* allele: 104 bp; floxed *Padi4* allele: 160 bp; knockout *Padi4* allele: 215 bp.

### Statistical analysis

Statistical significance was determined using Mann–Whitney or one-way ANOVA on Graphpad V 8 software.

## Supplementary Material

Supplementary information
