# Supplementary material for: Intrinsic function of the peptidylarginine deiminase PADI4 is dispensable for normal haematopoiesis
Source: Biol Open. 2022 Jun 13;11(6):bio059143. doi: 10.1242/bio.059143 (PMC9212077; doi:10.1242/bio.059143)
Supplement: Supplementary information [file biolopen-11-059143-s1.pdf]

Supplementary Figure 1

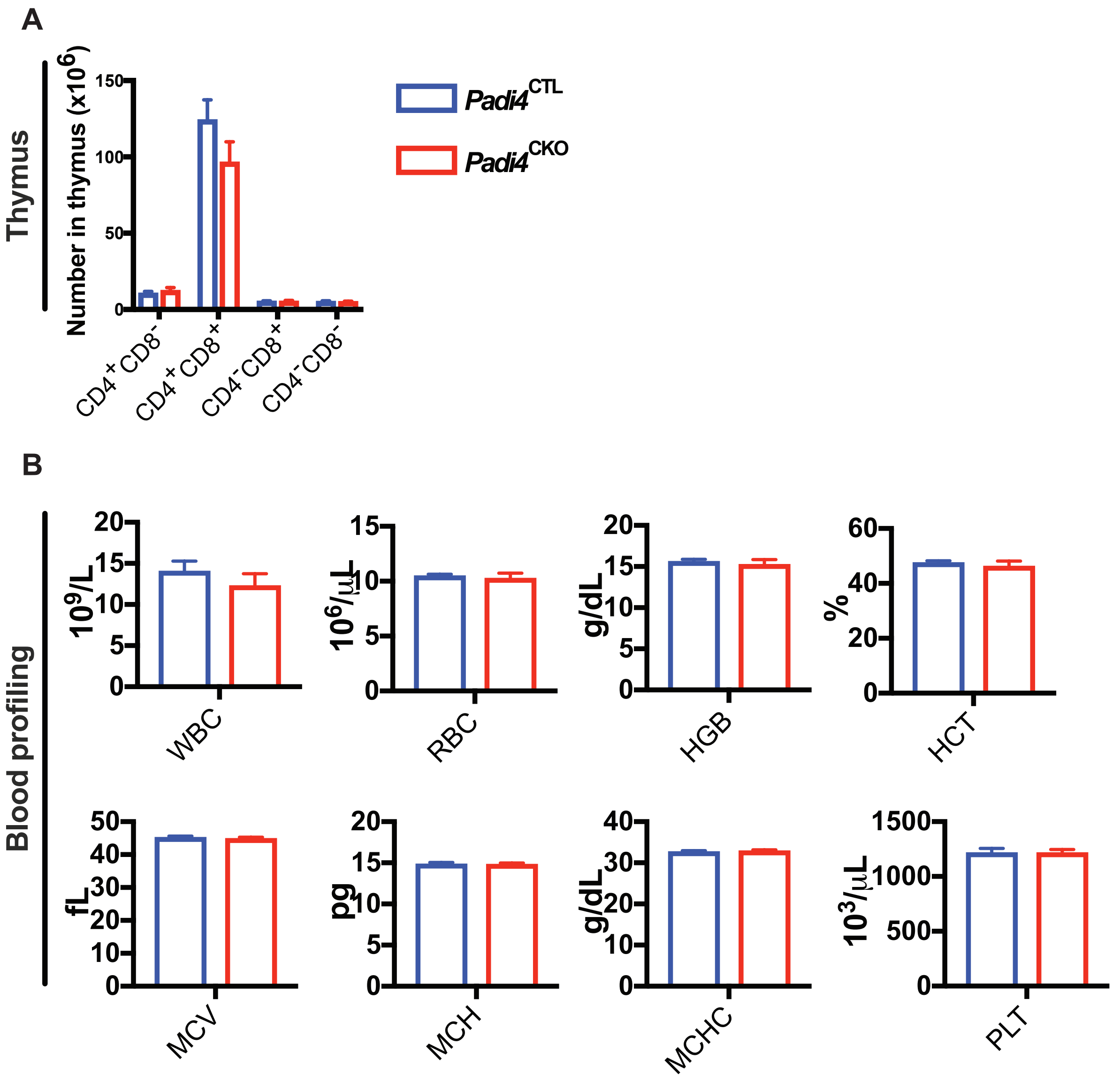

Fig. S1. Analysis of *Padi4* deletion in steady-state haematopoiesis. (A)Total number of thymic T cells. *Padi4*^{CTL}, n = 9; *Padi4*^{CKO}, n = 9. **(B)** Automated cell counting of blood samples from 8-12 week old *Padi4*^{CTL} and *Padi4*^{CKO}; WBC, RBC, HGB, HCT, MCV, MCH, MCHC and PLT counts. *Padi4*^{CTL}, n = 8; *Padi4*^{CKO}, n = 6. Data are mean ± SEM. *, P < 0.05; **, P < 0.01; ***, P < 0.001; ****, P < 0.0001 (Mann-Whitney U test).

Biology Open • Supplementary information

Supplementary Figure 2

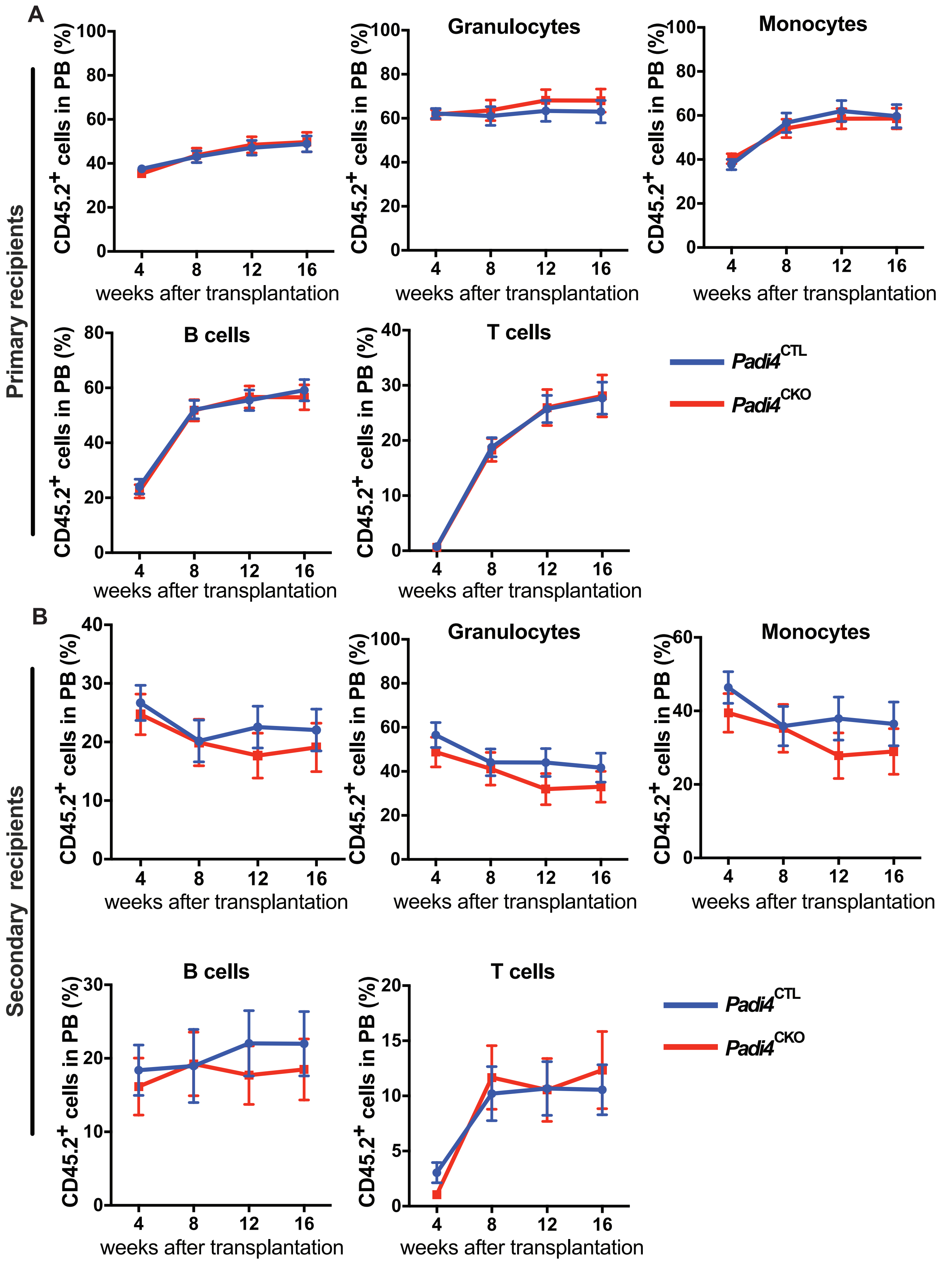

Biology Open • Supplementary information

Fig. S2. Peripheral blood analysis of mice transplanted with *Padi4*^{CTL} and *Padi4*^{CKO} bone marrow. Percentage of donor-derived CD45.2^{+} cells in PB and contribution of donor derived CD45.2^{+} cells to the Granulocyte, Monocyte, B cell and T cell population in PB. **(A)** Analysis of primary recipient mice. *Padi4*^{CTL}, n = 36; *Padi4*^{CKO}, n = 34. **(B)** Secondary recipient mice. *Padi4*^{CTL}, n = 21; *Padi4*^{CKO}, n = 22. Data are mean ± SEM. *, P < 0.05; **, P < 0.01; ***, P < 0.001; ****, P < 0.0001 (Mann-Whitney U test).

Supplmenentary Figure 3

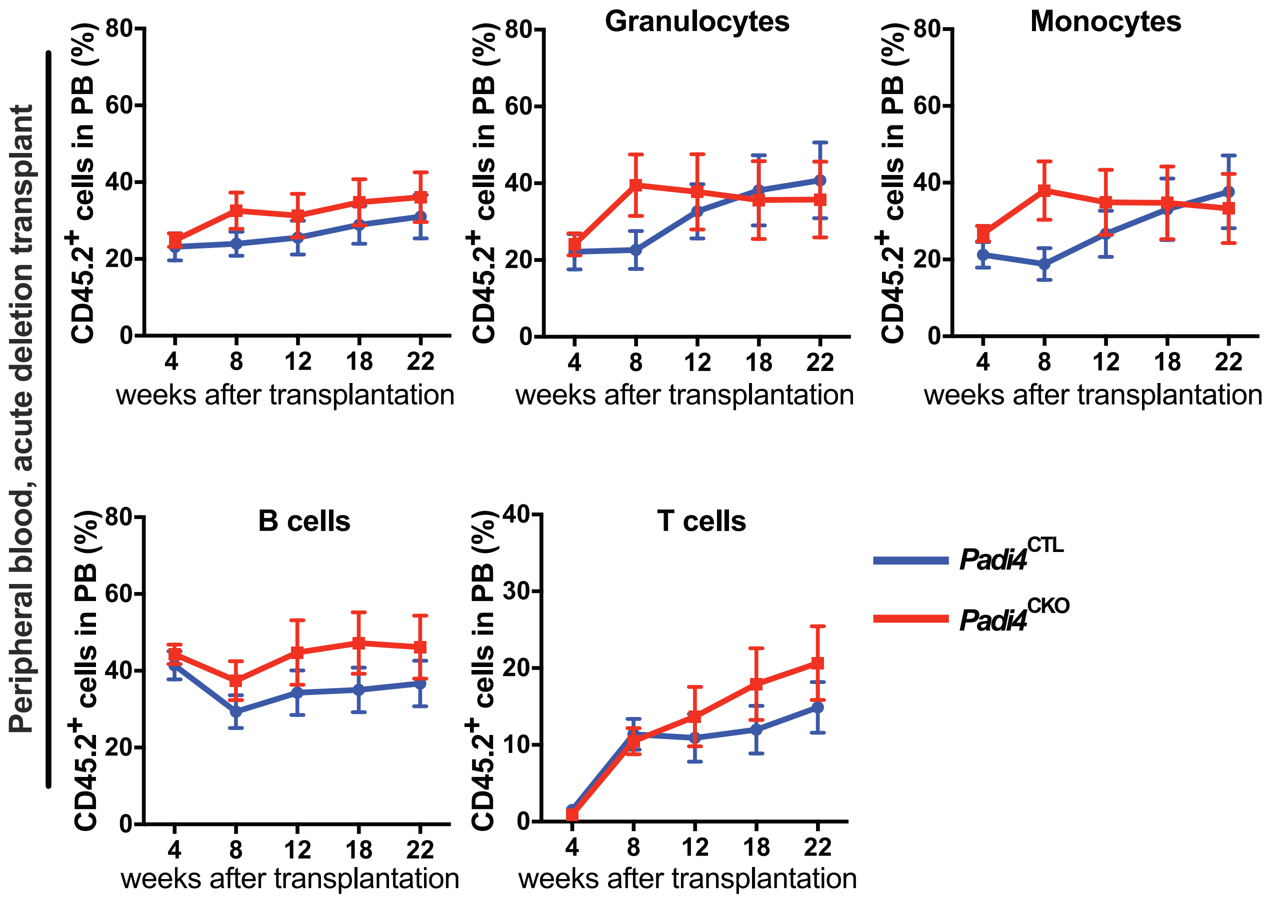

Fig. S3. Peripheral blood analysis after acute deletion of *Padi4*. Percentage of donor-derived CD45.2^{+} cells in PB and contribution of donor derived CD45.2^{+} cells to the Granulocyte, Monocyte, B cell and T cell population in PB of recipient mice, after acute deletion of *Padi4*. n = 15–21 recipients per genotype. n = 3-4 donors per genotype. Data are mean ± SEM. *, P < 0.05; **, P < 0.01; ***, P < 0.001; ****, P < 0.0001 (Mann-Whitney U test).

Biology Open • Supplementary information

Supplmenentary Figure 4

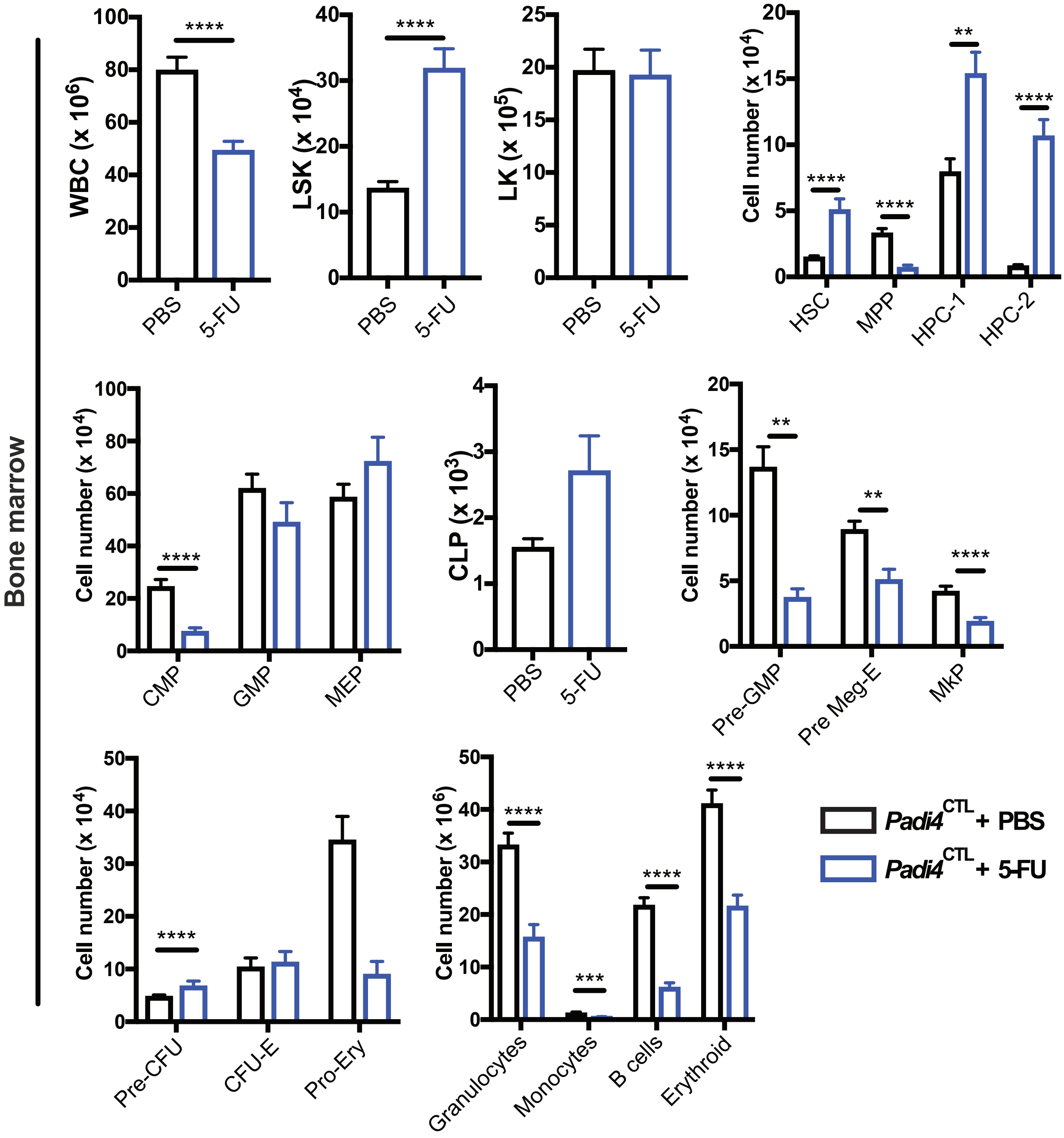

Fig. S4. 5-FU treatment leads to depletion of all bone marrow cells compartments in *Padi4*^{CTL} mice. Immunophenotypic analysis of *Padi4*^{CTL} mice treated with 5-FU or PBS vehicle control, demonstrating efficiency of 5-FU treatment. Experimental setup described in Figure 2A. Analysis was performed 10 days after the final dose of 5-FU; total number of cells in BM; WBC, LSK, and LK, HSC, MPP, HPC-1 and HPC-2 cells, myeloid, erythroid and lymphoid progenitor cells; CMP, GMP, MEP, CLP, Pre-GMP, Pre-MegE, MkP, Pre-CFU, CFU-E, Pro-Ery, differentiated Granulocytes, Monocytes, B cells, Erythroid cells and total number of cells in Spleen; WBC, B cells, Granulocytes and Monocytes *Padi4*^{CTL}, n = 21; *Padi4*^{CKO} n = 18. Data are mean ± SEM. *, P < 0.05; **, P < 0.01; ***, P < 0.001; ****, P < 0.0001 (Mann-Whitney U test).

Biology Open • Supplementary information

Supplmenentary Figure 5

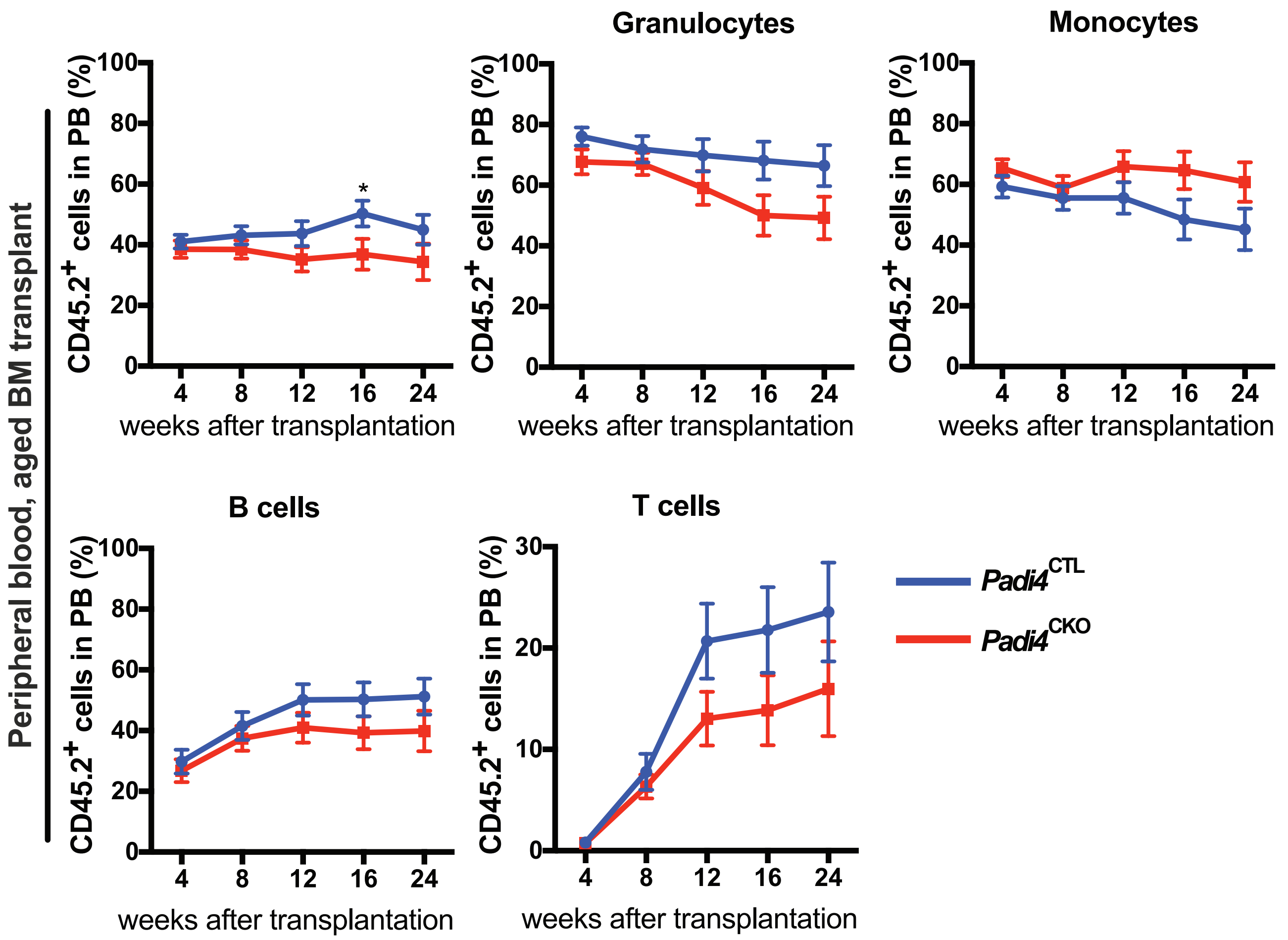

Fig. S5. Peripheral blood analysis of mice transplanted with aged *Padi4*[CTL] and *Padi4*[CKO] bone marrow. 200 CD45.2$^+$ BM HSCs from 1 year old mice were transplanted to primary recipient mice and monitored for 24 weeks following which immunophenotypic analysis was performed on BM and spleen. Percentage of donor-derived CD45.2$^+$ cells in PB. Contribution of donor derived CD45.2$^+$ cells to the Granulocyte, Monocyte, B cell and T cell population in PB. *Padi4*[CTL], n = 20; *Padi4*[CKO], n = 16. Data are mean ± SEM. *, P < 0.05; **, P < 0.01; ***, P < 0.001; ****, P < 0.0001 (Mann-Whitney U test).

Biology Open • Supplementary information

Table S1. List of antibodies used for flow cytometry. Antibody information for the analyses described in the Materials and Methods section *Flow Cytometry.*

Antibody	Conjugate	Catalog No.	Clone	Lot No.	Manufacturer
CD4	biotin	553649	H129.19	Various	BD Biosciences
CD5	biotin	553019	53-7.3	5062988	BD Biosciences
CD8a	biotin	553029	53-6.7	Various	BD Biosciences
CD11b	biotin	553309	M1/70	Various	BD Biosciences
CD45R/B220	biotin	553086	RA3-6B2	8127591	BD Biosciences
Ter119	biotin	553672	TER-119	Various	BD Biosciences
Gr-1/Ly-6G/C	biotin	553125	RB6-8C5	7275907	BD Biosciences
CD45.1	FITC	110706	A20	B202563	Biolegend
CD45.2	Pacific Blue	109820	104	B249623	Biolegend
Ter119	FITC	116206	TER-119	B272256	Biolegend
CD4	PE	130310	H129.19	B200770	Biolegend
CD48	PE	103406	HM48-1	B202873	Biolegend
CD150	PE-Cy7	115914	TC15-12F12.2	B238925	Biolegend
Gr-1/Ly-6G/C	PE-Cy7	108416	RB6-8C5	B209822	Biolegend
CD8a	APC	100712	53-6.7	B207080	Biolegend
CD8a	PE	100708	53-6.7	B223225	Biolegend
CD11b	Pacific Blue	101224	M1/70	B196387	Biolegend
CD11b	PE	101208	M1/70	B228654	Biolegend
CD11b	APC	101212	M1/70	B221810	Biolegend
CD117/c-Kit	APC	105812	2B8	B249344	Biolegend
CD117/c-Kit	BV-510	135119	ACK2	B209927	Biolegend
Sca-1/Ly-6A/E	FITC	122506	E13-161.7	B163258	Biolegend
Sca-1/Ly-6A/E	PE-Cy7	122514	E13-161.7	B194434	Biolegend
Sca-1/Ly-6A/E	Pacific Blue	122520	E13-161.7	B174209	Biolegend
CD19	APC-Cy7	115530	6D5		Biolegend
CD16/CD32	APC-Cy7	101328	93	B232340	Biolegend
CD71	PE	113808	R17217	B194428	Biolegend
CD127	BV-421	135023	A7R34	B241249	Biolegend
CD34	FITC	553733	RAM34	7341852	BD Biosciences
CD135	PE	553842	A2F10.1	8123884	BD Biosciences
CD41	APC	133914	MWReg30	B203704	Biolegend
CD105	PE	120408	MJ7/18	B169023	Biolegend
Streptavidin	PerCP	405213	–	B214631	Biolegend

Biology Open • Supplementary information
